# On the Assimilation of Instructions: Stimulus-response Associations are Implemented but not Stimulus-task Associations

**DOI:** 10.5334/joc.78

**Published:** 2019-07-31

**Authors:** Baptist Liefooghe, Frederick Verbruggen

**Affiliations:** 1 Department of Experimental Clinical and Health Psychology, Ghent University, BE; 2 Department of Experimental Psychology, Ghent University, BE

**Keywords:** Cognitive Control, Action, Attention

## Abstract

The assimilation of instructions consists of two stages. First, a task model is formed on the basis of instructions. Second, this model is implemented, resulting in highly accessible representations, which enable reflexive behavior that guides the application of instructions. Research frequently demonstrated that instructions can lead to automatic response activation, which indicates that stimulus-response associations can be implemented on the basis of a task model. However, instructions not only indicate how to respond (stimulus-response mappings) but also when (i.e., the conditions under which mappings apply). Accordingly, we tested whether instruction implementation leads both to the activation of stimulus-response associations and of associations between stimuli and the context or task in which the instructed stimulus-response mappings are relevant (i.e., stimulus-task associations). In four experiments, we measured if implementing newly instructed stimulus-response mappings also leads to bivalence costs (i.e., shorter latencies when a stimulus can only occur in one task compared to when it can occur in two tasks), which indicate the presence of stimulus-task associations. We consistently observed automatic response activation on the basis of instructions, but no bivalence costs. A discrepancy thus exists between information conveyed in an instructed task model and the elements of that task model that are implemented. We propose that future research on automatic effects of instructions should broaden its scope and focus both on the formation of an instructed task model and its subsequent implementation.

Humans have the ability to assimilate new tasks on the mere basis of instructions. Such assimilation presumably starts with an instruction phase (Brass, Liefooghe, Braem, & De Houwer, 2017), during which linguistic information is translated into a task model. For simple tasks, the construction of the task model only involves compiling a number of verbally instructed S-R mappings in an action-oriented format (Hartstra et al., 2012; Ruge and Wolfensteller, 2010). For more complex tasks (Bhandari and Duncan, 2014; Cole et al., 2013; Dumontheil et al., 2011), the construction of the task model is more complicated. The different sets of relevant rules need to be structured, which involves creating a hierarchical structure and information chunking (Bhandari & Duncan, 2014; Duncan et al., 1996, 2008; Verbruggen, McLaren, Pereg, & Meiran, 2018).

In order to result in overt behavior, a task model or its relevant parts need to become highly accessible, which is done in the implementation phase. Within the working-memory model of Oberauer (2009), an instructed task model is possibly represented in activated long-term memory and implementation consists of loading and maintaining parts of this model in a more activated layer of working memory, such as the bridge (Brass et al., 2017). The implemented representations then lead to “reflexive” behavior (Meiran, Liefooghe & De Houwer, 2017; Meiran, Cole & Braver, 2012). The latter hypothesis is supported by the observation that responses can be automatically triggered on the basis of newly instructed stimulus-response (S-R) mappings, which results in response-congruency effects. For instance, Liefooghe et al. (2012) introduced the inducer-diagnostic paradigm (see also Meiran et al., 2015 for the conceptually similar NEXT paradigm). On different runs of trials, participants are instructed with two new S-R mappings of an inducer task (e.g., If “X”, press left; if “Y”, press right), one of which needs to be applied when a green probe stimulus of the inducer task is presented. Before the mappings can be applied, a diagnostic task is performed, which shares stimuli and responses with the inducer task, but employs a different categorization rule (e.g., upright, press left; *italic*, press right). The diagnostic task is signaled by printing stimuli in black. Within such design, stimuli in the diagnostic task can thus be response congruent (e.g., “X” presented upright or “Y” presented in italic) or response incongruent (e.g., “Y” presented upright or “X” presented in italic), with the latter being associated with a performance cost compared to the former (i.e., *instruction-based congruency effect*; e.g., Braem et al., 2017; Everaert et al., 2014; Liefooghe et al., 2012, 2013, 2016; Theeuwes et al., 2014, 2015).

Although automatic effects of instructions have been extensively documented in recent years (see Brass et al., 2017; Meiran et al., 2017 for reviews), such research was mainly concerned with response-congruency effects and the implementation of S-R associations. However, the instructed task model, which underlies these implemented representations, may go beyond the mere representation of S-R associations. In order to perform a task in complex (multitasking) environments, instructed S-R mappings (i.e., how to respond) need to be represented as a function of the condition(s) under which these mappings apply (i.e., when to respond). For instance, when considering the inducer-diagnostic paradigm, participants would be unable to perform the inducer task, if they did not relate the instructed S-R mappings to a representation of that inducer task and when this task needs to be performed (e.g., when the stimulus is green in the aforementioned example). The efficient execution of a task, may thus not only require the implementation of S-R associations, but also the implementation of associations between stimuli and a representation of the context or task in which the implemented S-R mappings are relevant, which we will refer to as *stimulus-task associations*.^1^

First evidence suggesting that stimulus-task associations can be implemented on the basis of instructions was offered by studies investigating the extent by which instructions can lead to response-congruency effects and bivalence costs in task switching (Brass, Wenke, Spengler, & Waszak, 2009; Waszak, Wenke, & Brass, 2008). During task switching participants switch between two tasks, which typically share stimuli and responses (see Kiesel et al., 2010; Koch et al., 2018; Monsell, 2003; Vandierendonck, Liefooghe, & Verbruggen, 2010 for reviews). Stimuli are thus bivalent and afford the application of two tasks (e.g., a letter-number compound, ‘E9’, on which both a letter and number task can be applied). Bivalent stimuli can be response congruent (same response across different tasks; e.g. vowels and odd digits require a left response) or response incongruent (different responses across different tasks; e.g., vowels require a right response and odd digits require a left response). This contrasts with univalent stimuli, which only afford one task (e.g., ‘E#’). Rogers and Monsell (1995, see also Fagot, 1994) observed that responses to univalent stimuli were faster than responses to congruent bivalent stimuli, which in turn were faster than responses to incongruent bivalent stimuli. Similarly, Steinhauser and Hübner (2009) observed that latencies on univalent stimuli were shorter than the average latency on congruent and incongruent bivalent stimuli. In other words, besides a response-congruency effect (i.e., congruent vs. incongruent bivalent stimuli), indicating S-R associations, a bivalence cost is also present (i.e., univalent vs. bivalent stimuli): For bivalent stimuli, different stimulus-task associations compete, inducing an additional performance cost (i.e., the bivalence cost) compared to univalent stimuli, which are only related to one task (see also Monsell, Taylor, & Murphy, 2001; Rubin & Koch, 2006; Waszak, Hommel, & Allport, 2003, 2004; Woodward, Meier, & Tipper, 2003 for similar considerations).

Waszak et al. (2008; see also Brass et al., 2009) observed that bivalence costs can also be induced on the basis of merely instructed S-R mappings. In their studies, participants switched between a color and a shape task, which each consisted of four S-R mappings (e.g., color task: pink-left, red-left, yellow-right, blue-right; shape task: square-left, rectangle-left, triangle-right, star-right). Per task a subset of the S-R mappings was actually performed (e.g., color task: pink-left, yellow-right; shape task: square-left, triangle-right), whereas the other subset remained instructed but not executed (e.g., color task: red-left, blue-right; shape task: rectangle-left, star-right). In addition, stimuli could be bivalent by combining the target features of both tasks (e.g., a pink square) or univalent by combining a target feature with a completely irrelevant feature (e.g., a purple square). Waszak et al. (2008) observed that merely instructed S-R mappings induced a bivalence cost when being irrelevant (see also Brass et al., 2009), but no response-congruency effect.

The findings of Waszak et al. (2008) and Brass et al. (2009) suggest that the formation of a task model on the basis of instructions can subsequently lead to the implementation of stimulus-task associations. However, the absence of a response-congruency effect for instructed S-R mappings is at odds with the now frequent observation that responses can be automatically triggered on the basis of instructions (e.g., Braem et al., 2017; Everaert et al., 2014; Cohen-Kdoshay & Meiran, 2007, 2009; Liefooghe et al., 2012, 2013, 2016; Meiran et al., 2015; Theeuwes et al., 2014, 2015; Wenke et al., 2007, 2009, 2015). Liefooghe et al. (2012) argued that the absence of this effect in the task-switching studies of Waszak et al. (2008) and Brass et al. (2009) may have been the consequence of using eight different S-R mappings, which is highly demanding to implement. Such proposal corroborates with studies demonstrating that automatic effects of instructions are attenuated under high working-memory load conditions (e.g., Cohen-Kdoshay & Meiran, 2007; Meiran & Cohen-Kdoshay, 2012).

The inducer-diagnostic paradigm (Liefooghe et al., 2012) described above constitutes a sensibly less demanding approach to measure instruction implementation and the present study used this procedure to further investigate *which* elements of an instructed task model are implemented. In view of the assumption that instructed task models are hierarchical (Verbruggen et al., 2018), the central question was whether the implementation of the instructions of the inducer task not only involves making S-R associations highly accessible, but also stimulus-task associations. In order to investigate this issue, the diagnostic task included congruent and incongruent bivalent stimuli (see supra), as well as, univalent stimuli that were not assigned to a specific response by the instructions of the inducer task. Fueled by the findings of Waszak and colleagues, which indicated the presence of a bivalence cost, and the well-replicated observation of an instruction-based congruency effect in the inducer-diagnostic paradigm (e.g., Braem et al., 2017; Everaert et al., 2014; Liefooghe et al., 2012, 2013; Liefooghe & De Houwer, 2018), we hypothesized that both effects would co-occur, thus offering evidence that both S-R and stimulus-task associations are implemented.

## Experiments

**Figure 1:**
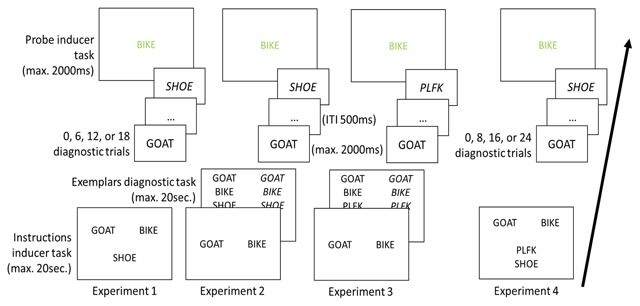
Schematic outline of the inducer-diagnostic paradigm used in Experiments 1–4.

We conducted four very similar experiments. In Experiment 1, each run started with the presentation of two new S-R mappings of the inducer task, each relating a noun to either a left or a right response. These S-R mappings were presented along with a third noun, which was not assigned to a particular response (see Figure 1). Participants were instructed that all three nouns would appear in the diagnostic task, but only the two nouns mapped to a specific response could appear in the inducer task. This was done to equate the familiarity of all stimuli and thus avoid potential orientation responses to the univalent stimuli. In Experiment 1, an instruction-based congruency effect was observed, but no bivalence cost. Concerned that the distinction between bivalent and univalent stimuli was not sufficiently clear in Experiment 1, S-R mappings of the inducer task and the possible exemplars of the diagnostic task were presented on separate instruction screens in Experiment 2. As such, we again emphasized via instruction that univalent stimuli could only be presented in the diagnostic task. Nevertheless, the same pattern of results was obtained. In Experiment 3, the distinction between univalent and bivalent stimuli was stressed even more by using different stimulus categories: the bivalent stimuli were nouns, whereas the univalent stimuli were randomly generated letter strings. Again the same results were obtained. In Experiment 4, we included both nouns and letter-strings as univalent stimuli to detect any potential differences in processing both types of univalent stimuli. Our results suggest this was not the case and we obtained a numerically similar pattern as in the previous experiments, namely an instruction-based response-congruency effect but no bivalence cost.

### Method

**Participants**. One-hundred-and-sixty-seven students at Ghent University participated in return for a 10 Euro payment (Experiments 1–3) or course credits (Experiment 4). Participants were naive to the purpose of the experiments and only participated in one of the four experiments (Experiment 1: n = 32; Experiment 2: n = 46; Experiment 3: n = 48; Experiment 4: n = 41). In each experiment the minimal sample size was n = 32, which ensures a power of .80 for detecting a medium-sized effect. Per experiment, 50 participation slots were made available and variations in the sample size of each experiment are the result of the number of participants available for testing at that time.

#### Experiment 1

***Stimuli & Materials***. S-R mappings and stimuli (words or letter strings) were presented in ARIAL font, size 16. Stimuli in the diagnostic task and the instructed S-R mappings of the inducer task were presented in black on a white background. Stimuli in the inducer task were presented in green. The A-key (left) and the P-key (right) of an AZERTY keyboard were used.

Both bivalent and univalent stimuli were nouns. To this end, a list of 144 four-letter nouns was generated by using the SUBTLEX-NL database (Keuleers, Brysbaert, & New, 2010). For each participant, 48 triplets of words were created randomly (one triplet per run; see below). Each triplet was assigned to one run of trials and used only once. Two nouns were randomly used to construct the two S-R mappings of the inducer task, by relating the identity of the noun to either a left or a right response. The third noun of each triplet was never presented as a target stimulus in the inducer task.

***Procedure***. Participants were tested in groups of two or three. Each participant was tested in a separate cubicle in which (s)he was placed in front of a 17-inch laptop with a keyboard attached to it. The experiment was programmed by using the Tscope library for C/C++ (Stevens, Lammertyn, Verbruggen, & Vandierendonck, 2006). At the start of the experiment, participants first signed an informed consent. Next, the overall instructions were presented and paraphrased if necessary (see below). The experiment consisted of a series of blocks; and each block consisted of a series of runs.

***Run structure*.** A schematic overview of the run structure is presented in Figure 1. Each run started with the presentation of the instructions of the inducer task. These consisted of two new S-R mappings that mapped a word stimulus to a specific response (e.g., CART-left: BIKE-right). The position of the S-R mappings was determined randomly, so that the S-R mapping referring to a certain response could be presented either above or below the screen center. During the instruction phase, a new univalent stimulus was also presented below the S-R mappings of the inducer task. Participants were instructed to press the space bar when they had processed the instructions.

Once the space bar was pressed, the diagnostic task started. In the diagnostic task, participants judged the orientation of target words (upright or *italic*), by pressing the same left or right response key that would be used in the upcoming inducer task. The left-right response assignment of the diagnostic task (i.e. upright-left/italic-right vs. upright-right/italic-left) was counterbalanced across participants and remained constant for each participant. 1/3 of the stimuli in the diagnostic task were congruent (i.e., the response required by the diagnostic task matched with the instructed response for the inducer task; e.g. when italic words required a right response, ‘*BIKE*’ would be a congruent trial), 1/3 were incongruent (i.e., the response required by the diagnostic task mismatched with the response instructed for the inducer task; e.g. ‘*CART*’), and 1/3 were univalent (i.e., the stimulus of the diagnostic task did not belong to the stimulus-set that was relevant to the inducer task; e.g. ‘*BIRD*’). On each diagnostic trial, a stimulus was presented for a maximum of 2000 ms or until participants responded. The stimulus offset was followed by a 500 ms inter-trial interval. The screen flashed red for 200 ms after incorrect or late responses.

Five-hundred milliseconds after the last diagnostic trial, the probe of the inducer task was presented. This involved the presentation of a single stimulus printed upright, for a maximum of 2000 ms. Participants had to respond to this probe based on the S-R mappings instructed at the onset of the run. Errors were followed by a 200 ms error feedback (i.e., the screen turning red). Following the inducer task, a 1500 ms inter-trial interval was inserted before the next run started by presenting a set of new instructions of the inducer task.

***Block structure***. The experiment started with a practice block, followed by four test blocks, with a small break after each block. Stimuli in the practice block were 36 given names (e.g., CARL, SARA,…) instead of nouns. Each practice and test block contained 12 runs, which could differ in length. Specifically, the diagnostic task could consist of either 0, 6, 12 or 18 trials. The different types of runs were presented in a random order. The length of the diagnostic task differed between runs to make the onset of the probe of the inducer task relatively unpredictable; this encourages participants to remain prepared for the inducer task while performing the diagnostic task (see also Liefooghe et al., 2012, 2013; Meiran et al., 2015).

In each run containing a diagnostic task, congruent, incongruent, and univalent stimuli were presented equally often (i.e., 1/3 of the trials). Taken together, 432 diagnostic test trials were administered with 144 trials of each stimulus type (congruent, incongruent, univalent). The experiment lasted for approximately 35 minutes.

***Instructions***. Participants were first informed that they would be presented with different runs of trials in which they had to perform two tasks. The first task that was introduced was the inducer task. Participants were informed that at the start of each run two new S-R mappings would be instructed. They had to encode these mappings, as one of the mappings would be probed at the end of the run. The instructions then stated that after pressing the spacebar these mappings would disappear and that one some runs they first had to complete a number of trials of the diagnostic task, which was then introduced and described. After a varying number of diagnostic trials, the probe of the inducer task would appear in green. These basic instructions were summarized on the next page of the instructions in which we also outlined the structure of the experiment (i.e., 1 practice block, 4 test blocks, number of runs in each block,…) and emphasized that both speed and accuracy were important. On this second instruction screen, we also mentioned that a third stimulus would appear below the mappings of the inducer task, but that this word would only appear in the diagnostic task and not in the inducer task. Following this second instruction screen, a schematic illustration of a run was provided. Participants could ask for additional explanations, if needed.^2^

#### Experiment 2

The only difference with Experiment 1 was the instruction phase. In Experiment 2, each run started with the presentation of two instruction screens. On the first screen, the two new S-R mappings of the inducer task were presented. After encoding these S-R mappings and pressing the spacebar, a second screen was presented in which the stimuli of the diagnostic task were presented. These included the two stimuli relevant in the inducer task, as well as the univalent stimulus. These stimuli were presented in two columns: Depending on the response assignment of the diagnostic task, upright stimuli were presented in a left column (e.g. *CART – BIKE – BIRD*) and italic stimuli in a right column (e.g. *CART – BIKE – BIRD*), or vice versa. After pressing the spacebar the diagnostic task started.

#### Experiment 3

The only difference between Experiments 2 and 3 was the nature of the univalent stimuli. More specifically, we used random strings of 4 consonants (e.g., “FKLP”) as univalent stimuli. For each run, a new string was generated and strings were never repeated across runs.

#### Experiment 4

In Experiment 4, two types of univalent stimuli were used: (a) ‘univalent nouns’ that were not part of the inducer instructions (like in Experiments 1–2); and (b) ‘univalent strings’ (like in in Experiment 3). Second, in order to have an equal number of observations for each stimulus type, 36 runs of trials were presented, which could either include 0, 8, 16, or 24 trials of the diagnostic task (9 runs per number of diagnostic trials). In the runs including trials of the diagnostic task, 1/4 was congruent, 1/4 incongruent, 1/4 comprised a univalent noun, and 1/4 comprised a univalent string. A total of 576 diagnostic trials was collected per participant. As such, we had 144 trials per stimulus type as in the previous experiments. This experiment lasted around 45 minutes.

Note that in Experiment 4, the instruction phase consisted of a single screen again (like in Experiment 1), as the comparison between Experiments 1–3 revealed that presenting the instructions on one or two instruction screens did not modulate our findings. The two S-R mappings of the inducer task were presented, with the univalent stimuli underneath.

**Data Analysis**. Raw data and corresponding processing scripts are deposited at https://osf.io/zspjq/. For each experiment, participants who fell below 2.5 standard deviation of the group mean accuracy of either the inducer or the diagnostic task were excluded. This led to the exclusion of nine participants. The final sample for each experiment was: Experiment 1: n = 31; Experiment 2: n = 43; Experiment 3: n = 46: and Experiment 4: n = 39. In addition, diagnostic trials were taken into account only on when the inducer task of the run was performed correctly (see also, Braem et al., 2017; Everaert et al., 2015; Liefooghe et al., 2012, 2013, 2016; Theeuwes et al., 2014; Verbruggen et al., 2018). For the analysis of the Reaction Time (RTs), only correct trials were considered. In addition, RT data-points higher than 2.5 standard deviation of each individual cell mean were discarded. These different restrictions led to the combined removal of 14.31% diagnostic trials in Experiment 1, 14.79% in Experiment 2, 12.36% in Experiment 3, and 18.96% in Experiment 4.

The analyses focused on the diagnostic task.^3^ RTs and error rates (ERs) of the diagnostic task were subjected to a one-way repeated measures ANOVA with Stimulus Type as a factor; this factor had three levels (congruent, univalent, incongruent) in Experiments 1–3, and four levels (congruent, univalent nouns, univalent strings, incongruent) in Experiment 4. All data processing and analyses were performed by using R (R Core Team, 2017). ANOVAs were calculated by using ‘afex’ (Singmann et al., 2018). Bayesian ANOVAs were also conducted with ‘BayesFactor’, using the default JZS prior (.707; Morey, Rouder, & Jamil, 2015). The Bayes factors (BF) denote *BF_1_* (i.e., the Bayes Factor for evidence in favor of the alternative hypothesis), and we interpret these using the classification discussed in Schönbrodt and Wagenmakers (2017; see also Jeffreys, 1961). Note that re-running the Bayesian ANOVAs may lead to slightly different results; this is due to sampling noise.

**Figure 2:**
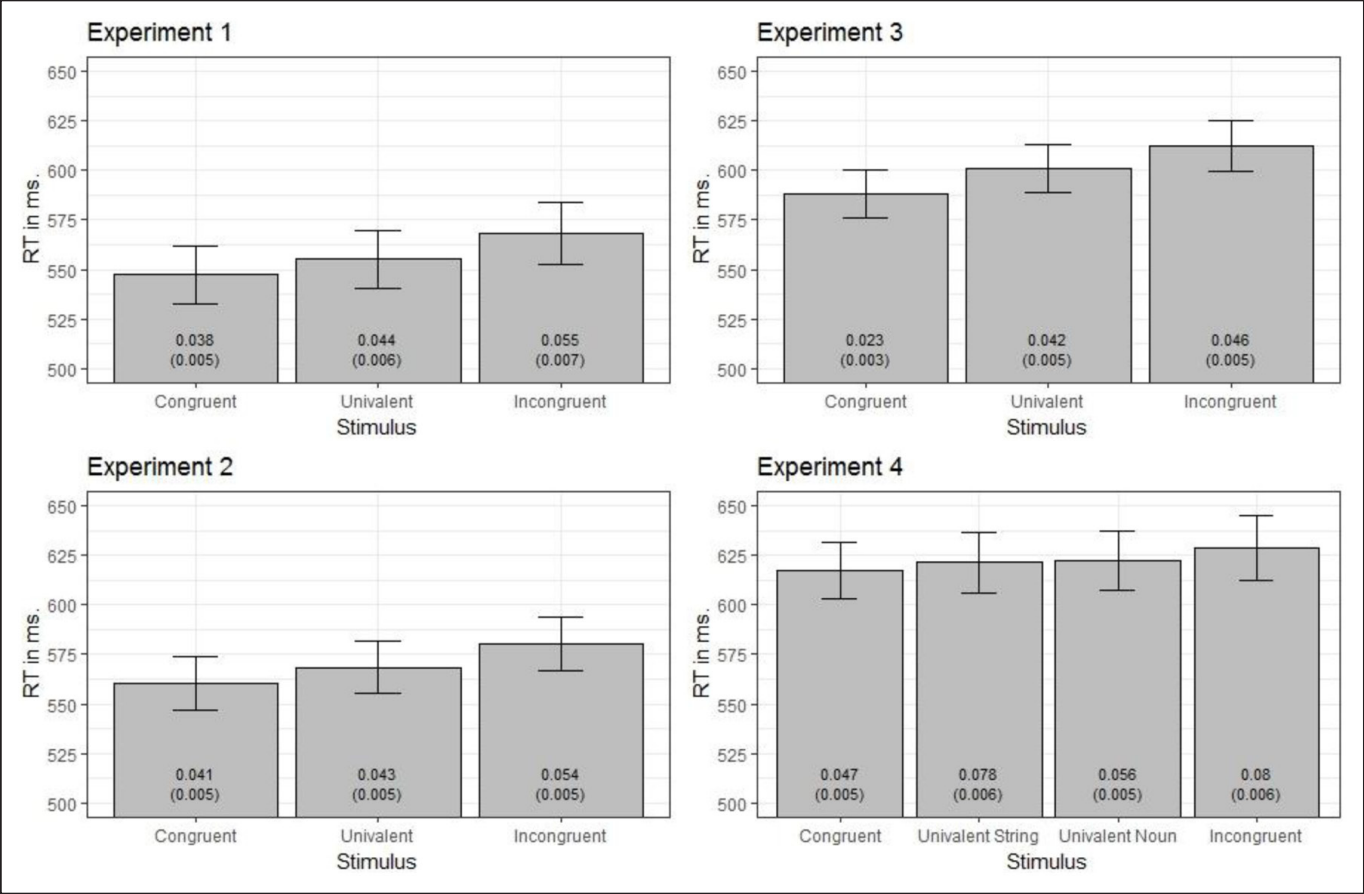
RTs (bars) and ERs (printed values) as a function of stimulus type in each experiment. Error bars denote standard errors. Standard errors for ERs are printed between brackets.

The outcomes of these analyses are reported in Table 1. An overview of the cell means and corresponding standard errors is presented in Figure 2. Finally, paired-sampled t-tests were used to decompose the main effects of Stimulus Type. For Experiments 1–3, these tests are presented in Table 2 and also include Bayesian t-tests (calculated with the default JZS prior; Morey, et al., 2015), effect sizes (Hedges’ *g*), and 95% confidence intervals. The contrasts conducted for Experiment 4 are presented in Table 3.

**Table 1:** Outcome of the ANOVAs conducted on the RTs and ERs of each experiment, as a function Stimulus Type.

		*DF*	*MSE*	*F*	*p*	*BF*	η^2^_p_

Experiment 1	RT	(2,60)	153.74	22.17	< 0.001	> 1000 ± 1.49%	.42
	ER	(2,60)	.0004	6.04	< 0.01	9.07 ± 0.71%	.17
Experiment 2	RT	(2,84)	213.53	19.63	< 0.001	> 1000 ± 0.89%	.32
	ER	(2,84)	.0005	4.18	< 0.05	2.20 ± 0.99%	.09
Experiment 3	RT	(2,90)	204.37	32.54	< 0.001	> 1000 ± 0.99%	.42
	ER	(2,90)	.0003	18.6	< 0.001	> 1000 ± 0.63%	.29
Experiment 4	RT	(3,114)	293.88	3.18	< 0.05	1.34 ± 0.52%	.08
	ER	(3,114)	.001	14.96	< 0.001	> 1000 ± 0.84%	.28

**Table 2:** Outcome of the paired t-tests used for decomposing the main effects of Experiments 1–3.

			Diff.	lower CI	upper CI	*DF*	*t*	*p*	*BF*	*g_av_*

Exp. 1	RT	Incongruent – Congruent	20.76	15.22	26.3	30	7.65	< .001	> 1000	.24
		Univalent – Congruent	7.79	0.88	14.72	30	2.3	< .05	1.86	.10
		Incongruent – Univalent	12.96	6.22	19.71	30	3.92	< .001	64.05	.15
	ER	Incongruent – Congruent	.02	0.01	.03	30	3.43	< .01	19.83	.54
		Univalent – Congruent	.01	.00	.02	30	1.40	.17	.46	.22
		Incongruent – Univalent	.01	.00	.02	30	2.03	.05	1.14	.29
Exp. 2	RT	Incongruent – Congruent	19.63	12.62	26.63	42	5.65	< .001	> 1000	.22
		Univalent – Congruent	7.91	2.56	13.26	42	2.98	< .01	7.59	.09
		Incongruent – Univalent	11.71	5.11	18.32	42	3.58	< .01	33.27	.13
	ER	Incongruent – Congruent	.01	.00	.02	42	2.49	< .05	2.59	.39
		Univalent – Congruent	.00	–.01	.01	42	.39	0.69	.18	.06
		Incongruent – Univalent	.01	.00	.02	42	2.46	< .05	2.42	.33
Exp. 3	RT	Incongruent – Congruent	24.04	17.95	30.19	45	7.95	< .001	> 1000	.28
		Univalent – Congruent	12.43	8.25	16.61	45	5.99	< .001	> 1000	.25
		Incongruent – Univalent	11.61	4.29	18.93	45	3.19	< .01	12.69	.14
	ER	Incongruent – Congruent	.02	.02	.03	45	5.99	< .001	> 1000	.87
		Univalent – Congruent	.02	.01	.03	45	4.72	< .001	908.21	.69
		Incongruent – Univalent	.00	–.01	.01	45	.89	0.38	.23	.11

**Table 3:** Outcome of the paired t-tests used for decomposing the main effects of Experiment 4.

		Diff.	lower CI	upper CI	*DF*	*t*	*p*	*BF*	*g_av_*

RT	Incongruent – Congruent	11.8	3.59	20.01	38	2.91	< .01	6.35	.12
	Un. String – Congruent	4.08	–2.86	11.02	38	1.19	.24	.33	.04
	Un. Noun – Congruent	5.32	–2.07	12.71	38	1.46	.15	.46	.06
	Incongruent – Un. String	–7.72	–16.06	.62	38	1.87	.07	.84	.08
	Incongruent – Un. Noun	–6.48	–15.69	2.74	38	1.42	.16	.44	.07
	Un. String – Un. Noun	–1.24	–8.01	5.53	38	.37	.71	.18	.01
ER	Incongruent – Congruent	.03	.02	.05	38	5.06	< .001	> 1000	.96
	Un. String – Congruent	.03	.02	.04	38	6.08	< .001	> 1000	.85
	Un. Noun – Congruent	.01	–.001	.02	38	1.75	.09	.69	.28
	Incongruent – Un. String	.002	–.01	.02	38	.33	.75	.18	.06
	Incongruent – Un. Noun	.02	.01	.04	38	4.02	< .001	101.47	.71
	Un. String – Un. Noun	.02	.01	.01	38	3.66	< .01	39.71	.60

### Results

#### Experiments 1–3

***Reaction times***. We first consider the RTs of the diagnostic task of Experiments 1–3, which are highly consistent. As can be seen in Table 1, the main effect of Stimulus Type was significant in each experiment. The contrasts (see Table 2) indicated longer RTs on incongruent stimuli compared to univalent stimuli and longer RTs on univalent stimuli compared to congruent stimuli. Thus, we observed the following RT pattern in Experiments 1–3: RT bivalent congruent < RT univalent < RT bivalent incongruent.

***Error rates***. When considering the ERs of Experiments 1–3, ERs were significantly higher on incongruent stimuli compared to congruent stimuli. However, the difference between incongruent and univalent stimuli was never significant and the difference between univalent and congruent stimuli was only significant in Experiment 3.

#### Experiment 4

***Reaction times***. The main effect of Stimulus Type was also significant in Experiment 4 (see Table 1). Mean RTs (see Figure 2) suggests a pattern that is in line with the previous experiments, namely, performance on the univalent stimuli being at an intermediate level compared to performance on congruent and incongruent stimuli. Further decomposition of this effect (see Table 3) indicated that RTs were significantly faster on congruent compared to incongruent stimuli. No other difference was significant. Furthermore, a Bayesian ANOVA of RTs only provided anecdotal evidence in support for the alternative hypothesis (i.e., an effect of Stimulus Type). In isolation, these findings should be interpreted with caution, but as noted above, the numerical pattern is consistent with the findings of Experiments 1–3.

***Error rates***. The effect of Stimulus Type was more pronounced for the ERs in Experiment 4. Fewer errors were made on congruent stimuli compared to incongruent stimuli. In addition, more errors were made on univalent strings compared to congruent stimuli and univalent nouns. ERs were only significantly higher on incongruent stimuli compared to univalent nouns but not in comparison to univalent strings.

#### Additional Analyses

***Different calculation of bivalence cost***. The bivalence cost can also be calculated by taking the difference between the mean performance on the congruent and incongruent stimuli (which are both bivalent) and the performance on the univalent trials (Brass et al., 2009; Steinhauser & Hübner, 2009; Waszak et al., 2008). These contrasts are presented in Table 4. In all four experiments, the difference between bivalent and univalent stimuli was not significant for the RTs, and Bayesian analyses provided (some) support for the null hypothesis. A similar pattern was present for the ERs when contrasting bivalent stimuli with univalent nouns (Experiments 1–2). But when contrasting bivalent stimuli with univalent strings in Experiments 3 and 4, anecdotal (Experiment 3) to moderate evidence (Experiment 4) was obtained, which indicated that ERs were slightly higher for the univalent strings compared to the mean error rate on the bivalent stimuli. Combined, these additional analyses also do not offer convincing evidence for the presence of a bivalence cost.

**Table 4:** Outcome of the paired t tests used to contrast average performance on congruent and incongruent bivalent stimuli with performance on univalent stimuli.

			Diff.	lower CI	upper CI	*DF*	*t*	*p*	*BF*	*g_av_*

Exp. 1	Bivalent – Univalent Noun	RT	1.66	–4.67	7.99	30	.54	.60	.22	.02
		ER	.00	–.01	0.01	30	–.46	.70	.21	.06
Exp. 2	Bivalent – Univalent Noun	RT	1.91	–2.99	6.79	42	.79	.44	.22	.02
		ER	.00	–.01	.00	42	–1.18	.24	.32	.15
Exp. 3	Bivalent – Univalent String	RT	.41	–5.54	4.71	45	.16	.87	.16	.01
		ER	.01	.00	.02	45	2.11	< .05	1.20	.26
Exp. 4	Bivalent – Univalent String	RT	1.82	–4.66	8.30	38	.57	.57	.20	.02
		ER	.01	.00	.02	38	2.79	< .01	4.89	.42
	Bivalent – Univalent Noun	RT	.58	–6.69	7.85	38	.16	.87	.18	.01
		ER	–0.01	–.02	.00	38	–1.73	.09	.68	.26

**Figure 3:**
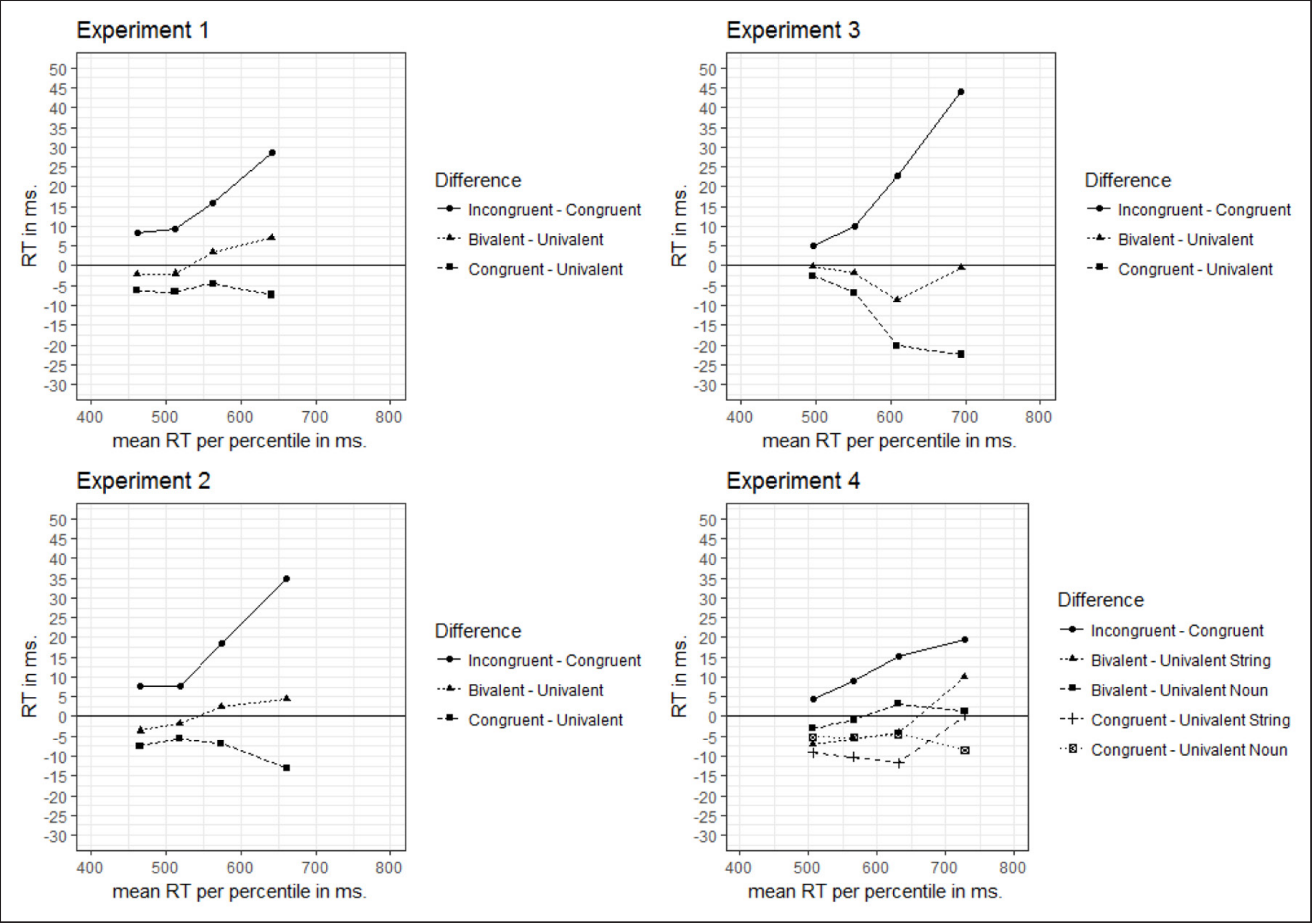
RT difference scores for the 20^th^, 40^th^, 60^th^ and 80^th^ percentile, between incongruent and congruent stimuli, congruent and univalent stimuli, and univalent and bivalent stimuli. X-axis values are the mean RTs across congruent, incongruent, and univalent stimuli per percentile.

***Distributional analyses***. Another possibility is that the instruction-based response congruency effect and the bivalence cost may occur on distinct parts of the RT distribution. Steinhauser and Hübner (2009) argued that bivalence costs are specifically present on the tail of the RT distribution, whereas response-congruency effects would be more likely to occur on fast responses (see also Heathcote, Popiel, & Mewhort, 1991; but see Roelofs & Piai, 2017; Matzke & Wagenmakers, 2009 for critical treatments of such dissociations). In view of these considerations, we plotted (a) the RT difference between incongruent and congruent stimuli; (b) the RT difference between congruent and univalent stimuli; and (c) the RT difference between bivalent (i.e., average between congruent and incongruent stimuli) and univalent stimuli, as a function of the 20^th^, 40^th^, 60^th^ and 80^th^ percentiles of the RT distribution (see also, Verbruggen et al., 2018 for a similar approach). These plots are presented in Figure 3. For all four experiments we observe that the difference between congruent and incongruent stimuli was more pronounced for the higher percentiles. More importantly, the difference between congruent and univalent stimuli remained negative in all four experiments, which indicates that RTs on univalent stimuli were always longer compared to RTs on congruent stimuli (including for slower responses). In addition, the difference between the mean performance on the bivalent stimuli and the univalent stimuli did not seem to increase drastically as a function of percentile.^4^

## Discussion

The present study further investigated, which elements of instructed task models are implemented into highly-accessible representations, which guide behavior. The rationale was that task models formed on the basis of instructions encompass more than S-R mappings and also include a representation of the condition under which these mappings apply. This hypothesis stems from previous studies suggesting that instructed task models are possibly hierarchical (Amso et al., 2014; Bhandari et al., 2017; Bunge & Zelazo, 2009; Chevalier, 2015; Verbruggen et al., 2018). Within this framework we reasoned that not only stimulus-response associations are implemented when assimilating new instructions, but also stimulus-task associations. We tested this idea in four highly similar experiments. Instruction-based response congruency effects served as a marker of implemented stimulus-task associations, whereas the bivalence cost served as a marker of implemented stimulus-task associations. Consistent with previous studies, we observed an instruction-based response-congruency effect in all experiments (see also, Braem et al., 2017; Everaert et al., 2014; Liefooghe et al., 2012, 2013; Liefooghe & De Houwer, 2018). However, despite having analyzed our data in several ways, we did not obtain any evidence in support of the presence of a bivalence cost: participants did not respond faster to univalent stimuli than to bivalent congruent stimuli or bivalent stimuli in general. Taken together, we obtained evidence for the implementation of S-R associations, but not for the implementation of stimulus-task associations. If stimulus-task associations are not implemented on the basis of instructions, the critical question becomes how participants proceed through a run of the inducer-diagnostic task and perform the relevant task at the appropriate time? Two scenario’s need to be considered: (a) a hierarchical model is formed on the basis of instructions, but not all its components are implemented; or (b) cue-compound strategies are used.

First, the possibility remains that a hierarchical task model is created on the basis of instructions, which includes the conditions under which the different rules apply, but that only parts of this task model are implemented. After all, Brass et al. (2017) argued that the implementation of a task model is demanding as it requires the maintenance of associations in a highly activated state (see also Meiran et al., 2012). Thus, the absence of automatic effects of instructions does not necessarily indicate that instructions were not fully translated into a task model. Consistent with this idea, Liefooghe et al. (2013) did not observe an instruction-based congruency effect when participants were not encouraged to prepare for the inducer task, despite the fact that they were still able to perform that inducer task. Similar findings were reported by Wenke et al. (2009), who argued that implementation is under strategic control and depending on the task context, participants may refrain to implement S-R associations in advance and only retrieve the correct response when the imperative stimulus is presented.

The nature of the elements of the instructed task model that are implemented might thus depend on the overall task demands. For example, in the task-switching studies of Brass et al. (2009) and Waszak et al. (2009), in which bivalence costs but no response-congruency effects were observed, participants had to alternate frequently and unpredictably between the different tasks. Participants thus first had to infer which task to perform on the basis of a task cue and subsequently decide which S-R mapping to apply to the target stimulus. In line with the proposals of Wenke et al. (2009), the strategy used by participants may involve the implementation of stimulus-task associations, such that the correct task could be quickly inferred. The relevant S-R association is then only implemented and applied in a later stage. In the inducer-diagnostic paradigm only one task switch is imposed in each run and the direction (albeit not the onset) of that switch (namely from the diagnostic task to the inducer task) is predictable. Considerably less demands are thus placed on the necessity to infer the correct task, and accordingly, it is less imperative to implement stimulus-task associations. In addition, the inducer-diagnostic paradigm requires the frequent assimilation of new S-R mappings. Accordingly, priority may have been given to the implementation of the corresponding S-R associations, as part of a preparatory strategy.

The second scenario that needs to be considered is that performance in the inducer-diagnostic paradigm is not underlain by a hierarchical task model. Within the field of task switching, Logan and Bundesen (2003, see also Schneider & Logan, 2005) argued that the seemingly complex activity of task switching does not necessarily require hierarchical control. In contrast, these authors conceptualized task-switching performance in terms of the *direct retrieval* of complex response rules without a distinction between different tasks. Applied in the current context, a flat or linear task model may be created, which includes the following complex rules: green + X + upright = > left; green + X + italic = > left; white + X + upright = > left; white + X + italic = > right; green + Y + upright = > right; green + Y + italic = > right; white + Y + upright = > left; white + Y + italic = > right; white + univalent stimulus + upright = > left; and white + univalent stimulus + italic = > right. As such, the correct response is retrieved on the basis of a combination of the color cues and the identity of the stimulus. However, two issues seem to argue against such a cue-compound explanation. First, cue-compound retrieval strategies seem highly artificial and are quickly overruled by strategies relying on hierarchical control (e.g., Dreisbach, Goschke, & Haider, 2007; Forrest, Monsell, & McLaren, 2014). Second, the presence of an instruction-based congruency effect in the absence of a bivalence cost in the current experiments indicates that no associations between stimulus and cue were implemented. This seems odd if participants retrieve responses on the basis of this specific combination of cues and stimuli.

At first sight, the present findings seem at odds with the observation that instructions can lead to the formation of stimulus-*category* associations (e.g., mouse-living; see Horner & Henson, 2009, 2011, 2012 for a discussion of this notion). This was evidenced in a variant of the item-specific priming paradigm (e.g., Moutsopoulou, Yang, Desantis, & Waszak, 2015), which was introduced by Pfeuffer and colleagues (Pfeuffer et al., 2017, 2018a, 2018b). Participants were presented with prime stimuli (e.g., mouse), which were accompanied with two verbal messages through a headphone: a category (e.g., living) and a response (e.g., left). The stimuli needed to be classified at a later stage either as being mechanical-living or as being small-large. In addition, the left-right response assignments to these categories varied. Probe stimuli could thus require a different categorization and/or response compared to the instructions presented together with the prime stimuli. Switching the category and/or the response between prime and probe triggered a performance cost in these studies (Pfeuffer et al., 2017, 2018a, 2018b), indicating that stimulus-response and stimulus-category associations can be formed on the mere basis of verbal messages. Interestingly, both performance costs do not interact, suggesting that stimulus-response and stimulus-category associations are formed independently. However, it is important to note that in these studies, the categories in the stimulus-category associations are *response* categories (e.g., living, mechanical, small, large) belonging to the classification tasks that need to be performed. In addition, stimuli and category have a strong preexisting semantic relation (e.g., mouse-living or mouse-small). In the present study, we investigated the formation of arbitrary associations between a stimulus and an abstract representation of the task in which an equally arbitrary S-R mapping was relevant. Thus, this task representation was not related to a particular response category, neither did it entertain a preexisting semantic relation with the stimulus. Finally, in the studies of Pfeuffer and colleagues, the relation between a stimulus and a response category was directly emphasized by means of a verbal message. In the present study, we directly instructed S-R mappings, but did not explicitly instruct stimulus-task mappings.

Based on the aforementioned discussion, the consideration needs to be made that the use of tasks in which stimuli are strongly related to a particular semantic category, could have mediated the presence of the bivalence cost observed in the studies of Waszak et al. (2008) and Brass et al. (2009). In both studies participants had to switch between a color- and a shape-judgment task. Although participants were instructed with arbitrary S-R mappings within each task (e.g., pink-left; square-right), both these tasks are based on distinct categories (colors and shapes) and stimuli have a strong sense of belonging to these categories. The tasks could thus easily have been conceptualized in terms of their underlying category. This may have facilitated the distinction between stimulus features, which were instructed to be potential targets and stimulus features that were not. As such, the distinction between bivalent stimuli (i.e., both stimulus features are related to a response) and univalent stimuli (only the relevant feature is related to a response) may also have been accentuated. By contrast, in the inducer-diagnostic procedure, the inducer task requires the classification of a word on the basis of its identity. Possibly, the inducer task was not conceptualized in terms of its underlying category of stimuli, namely words or nouns (e.g., the ‘word’-task). This is evidenced by the results of Experiment 4 where we did not observe consistent differences in processing univalent strings and univalent nouns during the diagnostic task. If the inducer task would have been related to the category ‘words’ or ‘nouns’, then univalent strings should have been processed in a faster pace than the univalent nouns, because the latter stimuli belong to the same category as the bivalent stimuli.

On a final note, it is interesting that the instruction-based congruency effect has a behavioral signature similar to the Stroop effect. In a Stroop task (Stroop, 1935; for reviews, see MacLeod, 1991, Kalanthroff, Davelaar, Henik, Goldfarb, & Usher, 2018), participants need to identify the print color of a color word while ignoring the meaning of the word itself (e.g., say “green” to the word “red” printed in green). The Stroop effect is the observation that participants are typically slower and less accurate in responding to incongruent trials (e.g., the word GREEN printed in yellow or GREEN_yellow_), where the meaning of the word and color mismatch, relative to congruent trials (e.g., GREEN_green_), where the meaning of the word and color match. Univalent stimuli can be created in a Stroop task by including distractors, which are not intrinsically related to one of the target colors (e.g. the word TABLE or the string XZKRD). Performance on such univalent stimuli is intermediate compared to performance on congruent and incongruent stimuli (i.e., RT congruent < RT univalent < RT incongruent). This pattern has been taken as evidence for the idea that the Stroop effect consists of a facilitation effect (RT univalent – RT congruent) and an interference effect (RT incongruent – RT univalent; see Augustinova & Ferrand, 2014; Augustinova et al., 2016 for reviews). A similar pattern was observed in the present study with respect to the instruction-based congruency effect (but see Meiran et al., 2015), which suggests that both response facilitation and response interference can be obtained on the basis of a newly instructed task, as it is the case for a well-trained task, such as word naming, which underlies the Stroop effect. This would again indicate that instructions can easily mimic effects of long-term practice (see also Theeuwes, et al., 2014 for similar considerations). An additional similarity between the instruction-based response-congruency effect and the Stroop effect is that both effects increase in size for slower responses. Such pattern of results may suggest that the information-accumulation processes underlying the Stroop effect and the instruction-based response-congruency effect are similar. For instance, Pratte et al. (2010) suggested that larger Stroop-effects on slower responses indicate that both stimulus dimensions (color and color word) contribute to the information-accumulation rate. As such, this rate is higher for congruent compared to incongruent stimuli. A similar scenario could also apply for the instruction-based response-congruency effect. However, other processes may induce similar effects. For instance, the irrelevant stimulus dimension may affect the response boundaries that are applied when making a decision about the relevant stimulus dimension. A detailed analysis of the instruction-based response-congruency effect is beyond the scope of the present study. However, it becomes clear that much may be gained by applying a more model-based analytic approach, for instance, by using Ratcliff’s diffusion model (Ratcliff, 1978).

Taken together, the present study set out to investigate whether the assimilation of new instructions involves the implementation of both S-R and stimulus-task associations. In contrast to what was previously reported (Brass et al., 2009; Waszak et al., 2008), we observed an instruction-based response-congruency effect but no instruction-based bivalence cost. This finding suggests that whereas instruction implementation leads to the formation of S-R associations it does not lead to the formation of stimulus-task associations. This is puzzling. After all, whether a task model is hierarchical or not (see supra), only part of that model – the relation between stimulus and response – seems to be implemented beforehand. Yet, implementation of S-R relations is not sufficient to explain task performance, as participants also need to know when to apply these rules. A discrepancy thus exists between information presumed to be represented in the task model, and the elements of that task model that are implemented. Understanding how instructions are assimilated and guide behavior, will require to investigate this gap. Future research on automatic effects of instructions will have to be conducted within this broader framework. In other words, what is instructed is not what is implemented and we need to understand why.

## Data Accessibility Statement

Raw data and corresponding processing scripts of all experiments are deposited at https://osf.io/zspjq/.

## Footnotes

In principle a more correct term would be stimulus-*response*-task association as one can assume that the whole stimulus-response association, and not only the stimulus, becomes related to a task. However, because stimulus-task association is more often used in the literature, we endorse the latter term.

Based on prior experience, we are aware that the inducer-diagnostic procedure is difficult at first. For this reason, in our studies we use an extensive practice phase, such that participants are familiar with all aspects of the design.

For completeness, we also checked performance on the inducer task: Experiment 1: mean RT = 723 ms (*SE* = 47), mean Error Rate or ER of .08 (*SE* = .05); Experiment 2: mean RT = 855 ms (*SE* = 66), mean ER = .10 (*SE* = .05); Experiment 3: mean RT = 912 ms (*SE* = 57), mean ER = .07 (*SE* = .04); and Experiment 4: mean RT = 886 ms (*SE* = 62), mean ER = .12 (*SE* = .05).

When considering the 80^th^ percentile this difference was 7.01 ms in Experiment 1, 4.42 ms in Experiment 2 and –.36 ms in Experiment 3. Additional contrasts indicated that these difference scores did not differ significantly from zero: Experiment 1, *t*(30) = 1.24, *p* = .23, *BF* = .38, *g_av_* = .44, 95% CI [–4.58, 18.61]; Experiment 2: *t*(42) = .99, *p* = .33, *BF* = .26, *g_av_* = .30, 95% CI [–4.55, 13.39], Experiment 3: *t*(45) = –.07, *p* = .95, *BF* = .16, *g_av_* = .02, 95% CI [–11.49, 10.76]. In Experiment 4 the difference between bivalent stimuli and univalent strings was 10.04 ms and 1.33 ms between bivalent stimuli and univalent nouns. Contrasts again indicated that these difference scores did not differ significantly from zero: *t*(38) = 1.58, *p* = .12, *BF* = .53, *g_av_* = .50, 95% CI [–2.88, 22.96] and *t*(38) = .19, *p* = .85, *BF* = .18, *g_av_* = .06, 95% CI [–13.04, 15.71].

## Author Information

BL was supported by grant BOF16/MET_V/002 of Ghent University. FV has received funding from the European Research Council (ERC) under the European Union’s Horizon 2020 research and innovation program (grant agreement No 769595). The authors are indebted to Jan De Houwer for his support and Marc Brysbaert for kindly providing credit students for Experiment 4. Correspondence concerning this article should be addressed to Baptist Liefooghe, H. Dunantlaan 2, B–9000, Ghent, Belgium. E-mail: baptist.liefooghe@ugent.be.
